# Selection and validation of reference genes for qRT-PCR analysis during biological invasions: The thermal adaptability of *Bemisia tabaci* MED

**DOI:** 10.1371/journal.pone.0173821

**Published:** 2017-03-21

**Authors:** Tian-Mei Dai, Zhi-Chuang Lü, Wan-Xue Liu, Fang-Hao Wan

**Affiliations:** 1 State Key Laboratory for Biology of Plant Diseases and Insect Pests, Institute of Plant Protection, Chinese Academy of Agricultural Sciences, Beijing, PR China; 2 Agricultural Genome Institute at Shenzhen, Chinese Academy of Agricultural Sciences, Shenzhen, PR China; Beijing Forestry University, CHINA

## Abstract

The *Bemisia tabaci* Mediterranean (MED) cryptic species has been rapidly invading to most parts of the world owing to its strong ecological adaptability, which is considered as a model insect for stress tolerance studies under rapidly changing environments. Selection of a suitable reference gene for quantitative stress-responsive gene expression analysis based on qRT-PCR is critical for elaborating the molecular mechanisms of thermotolerance. To obtain accurate and reliable normalization data in MED, eight candidate reference genes (*β-act*, *GAPDH*, *β-tub*, *EF1-α*, *GST*, *18S*, *RPL13A* and *α-tub)* were examined under various thermal stresses for varied time periods by using geNorm, NormFinder and BestKeeper algorithms, respectively. Our results revealed that *β-tub* and *EF1-α* were the best reference genes across all sample sets. On the other hand, *18S* and *GADPH* showed the least stability for all the samples studied. *β-act* was proved to be highly stable only in case of short-term thermal stresses. To our knowledge this was the first comprehensive report on validation of reference genes under varying temperature stresses in MED. The study could expedite particular discovery of thermotolerance genes in MED. Further, the present results can form the basis of further research on suitable reference genes in this invasive insect and will facilitate transcript profiling in other invasive insects.

## Introduction

Global change and climate warming have exposed ectothermic organisms to multifarious environmental stresses, which in turn allow organisms to use or exploit various strategies to increase their adaptability in face of environmental changes [1, 2]. Invasive insects, with the characteristics of spread rapidly and colonized successfully throughout varying environments, are becoming optimal models for studying the patterns and mechanisms of adaptability to environmental variation [3, 4]. Temperature is one of the foremost environmental factors affecting the rate of insect invasions as well as the abundance, distribution on a global scale [5–7]. With the mean global temperature increasing, exotic insects are exhibiting an upper thermal limit [8–10]. Understanding the thermal stress response patterns could be helpful in revealing the invasion mechanism of insects’ rapid adaptation to the new environments.

Gene expression data associate with the main characteristics of the rapidly response of stress-responsive during biological invasion is crucial to study the insects expansion and adaptation mechanisms [2, 11]. At the transcriptional level, reverse transcription quantitative polymerase chain reaction (qRT-PCR) has currently become a valuable tool for assessing gene expression and quantifying mRNA transcripts [12, 13] owing to its high sensitivity, accuracy, specificity, reproducibility and synchronized quantification of gene expression in diverse samples with a broad dynamic range [14]. However, its accuracy and reliability are determined by several experimental variations such as quality and amount of initial sample, RNA integrity, cDNA quality and PCR amplification efficiency [15]. In order to overcome the limitation of this method, a robust normalization strategy is utilized by using reference genes, whose expression levels should remain stable independent of tissue types, developmental stages and experimental conditions [16, 17]. Nevertheless, previous experiments evidently showed that the expression of universal reference genes in arthropods could be variable, particularly in different tissues, developmental stages and experimental conditions such as length of exposure to distinct abiotic factors [18–21]. Moreover, normalisation against a single reference gene has been found to be insufficient without the selection of multiple reference genes [15, 22, 23]. Consequently, for accurate normalisation it is vital to evaluate the stability of reference genes for each species and experimental designs prior to qRT-PCR analysis [24, 25].

The whitefly *B*. *tabaci* (Gennadius) (Hemiptera: Aleyrodidae) is a cosmopolitan, polyphagous invasive and economically important pest with a wide range of crops through direct feeding, depositing honeydew and transmitting plant viruses [26–28]. MED, which was first characterized in samples collected in the south of Spain and Portugal [29], has successfully invaded and colonized to most of the provinces. Subsequently, MED could be fast adaptation with a wide range of dramatically different local environments in China and widely displace local cryptic species and Middle East-Asia Minor 1 (MEAM1) [30], owing to its potential to adapt to various environmental temperatures [31–33]. *B*. *tabaci* has emerged as a promising model to understand the molecular basis of abiotic stress tolerance and adaptation for invasion biology [34–37]. As an invasive species, thermotolerance represent crucial mechanism for MED to successfully colonize new habitats [38–40]. Thus, the knowledge of MED temperature tolerance at the molecular level is invaluable for understanding its biological and invasive traits.

Till date, the study about using suitable reference genes for gene expression analysis under different thermal stresses for varied range of time periods was not comprehensive in MED. The present study was carried out to assess the most suitable candidate reference genes for normalisation of qRT-PCR data in MED after exposed to cold and heat temperature in a short- and long-term basis experiments. Eight reference genes, which commonly used as reference genes in previous studies were selected for assessment, including *18S ribosomal RNA* (*18S*), *glutathione S-transferase* (*GST*), *beta-actin* (*β-act*), *glyceraldehyde 3-phosphate dehydrogenase* (*GAPDH*), *beta-1-tubulin* (*β-tub*), *alpha-tubulin* (*α-tub*), *ribosomal protein L13a* (*RPL13A*) *and elongation factor 1 alpha* (*EF1-α*). Analysis was performed by using the three MS Excel-based programs including GeNorm [24], NormFinder [41] and BestKeeper [22]. To validate the selected reference genes, the expression profile of target gene *transient receptor potential (TRP)* was investigated.

## Materials and methods

### Insects

MED population was collected from tomato plants in Beijing (40°1′ N, 116°6′ E), China in July in 2012, and had been maintained in the laboratory for 50-60 generations when used in the experiments. MED was maintained, without laboratory exposure to insecticides, on tomato plants, *Lycopersicon esculentum* Mill (Zhongza No. 9), reared at 26 ± 2°C with 50 ± 10.0% relative humidity (RH) and a 14 L:10D photoregime provided by artificial lights. All tests were performed with adults.

### Treatments

Considering that MED had invaded to almost all the provinces striding across latitude 3°51′ N- 53°33′ N from south to north [42], and emerged in the field from mid-April to late October [43–45]. During above periods, the lowest and highest temperature of different regions ranges from 0-20°C and 15-40°C, respectively (https://www.wunderground.com/history/). Thus, based on the occurrence time of MED in filed, we designed a serial of temperatures for short-term thermal stresses, including cold temperatures (0°C, 12°C) or heat temperatures (35°C, 40°C) for varied time periods (1 h, 3 h, 5 h). Simultaneously, considering the optimal temperature of MED ranges from 28°C to 33°C, and the development extreme temperature range is 15–35°C [46–50], as well as mean monthly temperatures of different regions range from 15-35°C, we designed a serial of temperatures for long-term thermal stresses, including cold temperatures (17°C, 21°C) or heat temperatures (32°C, 35°C) for varied time periods (5 d, 10 d, 15 d, 20 d and the time of G0 to G1). G1 means the time that G0 young adults were exposed to thermal stress to spawn until hatch into G1 young adults. The stability of candidate reference genes was tested for adult whitefly across above thermal stresses. Because adult age is associated with different responses to temperature stress [51], we standardized adult age using only newly emerged whitefly adults that were younger than 3 hours. The ratio of female-male was 1:1 in each treatment group.

### Short-term thermal stresses

Newly emerged MED adults were placed together in a 5 mL centrifuge tube. To confirm all whitefly adults underwent temperature stress, the tubes were covered with cotton along 1/4 tube length from the top of the tubes. The whiteflies inside the tubes were exposed to cold temperatures (0°C, 12°C) in three different durations (1 h, 3 h, 5 h) in a constant environment (K6-cc-NR; Huber Kältemaschinenbau GmbH, Offenburg, Germany) and heat temperatures (35°C, 40°C) in three different durations (1 h, 3 h, 5 h) in a water bath (CC-106A; Huber Kältemaschinenbau GmbH, Offenburg, Germany), respectively. After stress exposure, all samples were frozen immediately in liquid nitrogen for 3 min, then stored at -80°C prior to qRT-PCR analysis. Adults maintained at 26°C (1 h, 3 h, 5 h) were used as untreated controls. Each treatment had three repetitions and each repetition had 200 adults.

### Long-term thermal stresses

Newly emerged MED adults were collected from tomato plants for each of the follow four treatments, which were conducted in climate-controlled chambers (Shaifu, Ningbo, China) at a photoperiod of 14:10 (L: D) h, and 65 ± 10.0% RH. The whiteflies inside the cages were exposed to cold temperatures (17°C, 21°C) for 5 d, 10 d, 15 d, 20 d and G1, and heat temperatures (32°C, 35°C) for 5 d, 10 d, 15 d, 20 d and G1, respectively. After stress exposure, all samples were frozen immediately in liquid nitrogen for 3min, then stored at -80°C prior to qRT-PCR analysis. Adults maintained at 26°C (5 d, 10 d, 15 d, 20 d, G1) were used as untreated controls. Each treatment had three repetitions and each repetition had 200 adults.

### Candidate reference genes and primers design

In the present study, four candidate reference genes (*β-act*, *GAPDH*, *β-tub*, *EF1-α)* were chosen due to their highest expression stability under temperature stresses [52–55]. For examples, *EF1-α* and *GAPDH* were the most stable reference genes under various abiotic stresses (included thermo stresses) in *Panonychus citri* [55], *β-act* and *GAPDH* could be the reference genes in *Channa striatus* under both short and long-term thermal stresses [52], *β-tub* was the most stable genes during thermal stresses in *Toxoptera citricida* [53]. The other four performed reference genes (*18S*, *GST*, *α-tub* and *RPL13A*) were selected since they had revealed stable expression patterns in different developmental stages and tissues in *B*. *tabaci* [21], *Rhodnius prolixus*[56], *Bactrocera minax* [57], *Thitarodes armoricanus* [58]. Traditionally, these genes are involved in ubiquitous cellular processes and commonly used as internal controls for qRT-PCR in hemiptera [19, 59, 60]. The primers used in the qRT-PCR reactions were designed with Oligo 7.0 software (MBI Inc, Cascade, CO, USA). The main characteristics of the primers are listed in Table 1.

**Table 1:** Candidate reference genes and primers used for qRT-PCR analysis.

Gene name	Primer sequences(F/R)	Amplicon length (bp)	Tm(°C)	R^2^	Efficiecy(%)
*18S*	AAACGGCTACCACATCCAAG	144	60	0.993	110.074
	GTCCTCGTCGCCTTGTTTAC				
*β-actin*	TCACCACCACAGCTGAGAGA	230	60	0.999	100.248
	CTCGTGGATACCGCAAGATT				
*GAPDH*	CTTGAGAGCCTCCTTGGAAC	104	60	0.999	100.391
	CGTGGGTGGAATCATACTTG				
*GST*	TTCGCCAGCTATACCTGATTT	99	60	0.997	103.300
	TTTGGGAAACTGCCAATCTT				
*α-tub*	ATTCACCTCCCTTCTCATGG	150	60	1	102.770
	GAGTGTTCCAAGGTGGTGTG				
*EF1-α*	TAGCCTTGTGCCAATTTCCG	110	60	0.998	101.136
	CCTTCAGCATTACCGTCC				
*β-tub*	TGTCAGGAGTAACGACGTGTTTG	167	60	0.990	106.322
	TTCGGGAACGGTAAGTGCTC				
*RPL13A*	CATTCCACTACAGAGCTCCA	101	60	0.996	101.858
	TTTCAGGTTTCGGATGGCTT				
*TRP*	GAAGACACCGAGCGTGGACAAAG	217	60	0.992	107.126
	GGCAATAGCGTTCCAGTCCTTTT				

Tm, melting temperature. R^2^, coefficient of determination.

### RNA extraction and cDNA synthesis

Total RNA was extracted from approximately 200 MED adults using the Trizol reagent (Invitrogen, Carlsbad, CA, USA) following the manufacturer’s protocol. The RNAs were quantified via NanoPhotometer^TM^ P330 (Implen, Munich, Germany), and with A260/A280 ratios were ranging from 1.8 to 2.0 and A260/A230 ratios were typically above 2.0. The RNA quality was also evaluated via 1% agarose gel electrophoresis. 2.0 μg of total RNA was used to synthesize cDNAs using the Super Script First-Strand Synthesis System (Transgen, Beijing, China) according to the instructions.

### Quantitative real-time PCR

Real-time PCR were performed using an ABI 7500 Real-time PCR system (Applied Biosystems, Foster, CA, USA). All the amplifications were confirmed by sequencing, and the specificity of qRT-PCR reactions was estimated by melting curve analysis. For qRT-PCR assays, 10 μL of 2×TransStart^TM^ Green qPCR SuperMix (Transgen, Beijing, China) were used for reaction mixtures that also contained 1 μL of the cDNA template, 0.4 μL of Passive Reference Dye, and 0.3 μL of each 10 μM primer solution in a 20 μL final volume. A thermalcycler was with the following cycling condition: (1) 30 s at 94°C, (2) 40 cycles of 5 s at 94°C, 30 s at 60°C. Three independent biological duplications were performed for all the reference genes studied and data for each biological duplicate were carried out in triplicate. A control without cDNA template was included in all of the batches. The amplification efficiency (E) of all primer pairs was validated by constructing a standard curve using a 2-fold serial dilution of cDNA template using the equation: E = (10^[-1/slope]^-1) ×100 [61].

### Statistical analysis

Expression levels were determined as the number of cycles needed for the amplification to reach a fixed threshold in the exponential phase of the PCR reaction [62]. Gene stabilities of the eight candidate reference genes were evaluated by three commonly used software, BestKeeper, geNorm version 3.5 (https://genorm.cmgg.be/), and NormFinder version 0.953 (http://moma.dk/normfinder-software/). GeNorm is one of the most commonly used algorithms to estimates the expression stability measure (M-value) calculating the mean pairwise variation (V) of each gene relative to all other genes included in the analysis [24]. Gene with the lowest M-value is considered the most stable gene in the tested conditions. In addition, to determine the optimal number of genes reference genes to use, the pairwise variation Vn/n+1 can be calculated between two sequential normalization factors. A large pairwise variation means that the added reference gene has a significant effect and should preferably be included for producing a reliable normalization factor. NormFinder is another Excel-based visual basic application that uses a model based on a variance estimation approach analyzing each sample set individually and also estimating intra- and inter-group variation in expression across different sample sets [41]. Top ranked reference genes will have minimum intra group variation and the lowest stability value. BestKeeper estimates the variability in the expression of the reference genes based on calculations of the Ct data variations and pairwise coefficient of variance of expression levels and identifies the best genes as those with least standard deviation (SD). For both geNorm and NormFinder, Ct values are necessary to convert into relative quantities using the formula: 2^−ΔCt^, in which ΔCt = the corresponding Ct value−minimum Ct value according to the described methods. For the BestKeeper application, the raw averaged Ct values were used (without transformation) to calculate the gene stability values according to the instructions contained with the program. Statistical analyses of the gene expression data were determined by ANOVA in SPSS v.16.0 (SPSS Inc, Chicago, IL, USA) with a threshold of P < 0.05.

### Estimation of expression of *TRP* gene

In order to validate the selected reference genes, we analyzed the relative expression levels of TRP gene. To compare the influence of different normalization strategies, the expression of TRP genes was normalized using both selected reference gene individually and gene combinations using the 2^−ΔΔCt^ method (Livak & Schmittgen) [63]. Based on the equation ΔΔCt = (Ct_target_ − Ct_reference_) treatment − (Ct_target_ − Ct_reference_) control. A 26°C sample was used as the calibrator sample (expression = 1). Target gene mRNA expression were analysed using one-way ANOVA followed by Fisher’s least significant difference (LSD) test with a threshold of P < 0.05.

## Results

### Selection of candidate reference genes, primer specificity and amplification efficiency

Seven reference genes primer sequences used in this study were previously identified in *B*. *tabaci* [18, 21]. While, primer sequences of *β-tub* were designed from the conserved regions of related sequences available in the transcriptome of *B*. *tabaci* [64]. The specificity and accuracy of the primers were confirmed by melting curve analysis (S1 Fig), where single amplification and single peak suggested the absence of primer dimmers and non-specific PCR products, respectively. The E and correlation coefficients (R^2^) of the candidate reference genes were determined using the slopes of the standard curves obtained by serial dilutions which fall in the acceptable range of 100–110% for the E, and 0.99–1.00 for the R^2^. The information of qRT-PCR primers and the amplification characteristic of reference genes are summarizes in Table 1.

### Expression profiles of reference genes during thermal stresses

**Fig 1:**
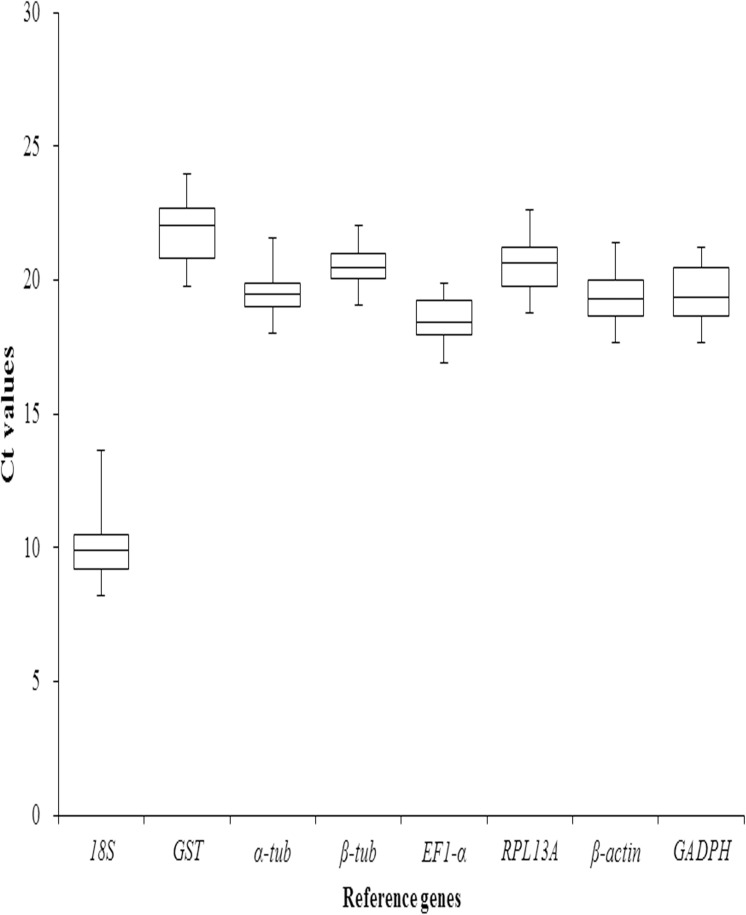
Expression levels of eight candidate reference genes in MED. Line across the box depict the median value and inside the box show the Ct values. The top and bottom whiskers are determine by the 25^th^ and 75^th^ percentiles, respectively.

The raw expression levels for eight reference genes were determined across all sample sets and represented in Tables A-D in S1 File. Means and standard deviations of Ct values for eight reference genes are summarized (Table 2; Fig 1). Gene expression analysis of eight reference genes across the 40 samples exhibited a wide range of mean Ct value, ranging from 9.94 for *18S* to 22.40 for *GST*, while the mean Ct of the majority of reference genes ranged from 18.57 to 20.75 with moderately abundant expression level. In the five groups, the mean Ct values showed a minimum of 9.41±1.55 and maximum of 22.90±0.93 for highest and lowest expression levels for *18S* and *GST*, respectively (Table 2).

**Table 2:** Distribution of the Ct values of each candidate reference gene in *B*.*tabaci* MED.

Gene name	All samples Ct±SD	Short-term cold exposure Ct±SD	Short-term heat exposure Ct±SD	Long-term cold exposure Ct±SD	Long-term heat exposure Ct±SD	Control Ct±SD
*18S*	10.25±1.35	10.11±0.92	9.58±0.94	9.41±1.55	11.34±1.77	10.56±0.76
*GST*	22.11±1.40	22.90±0.93	21.58±1.05	20.95±1.60	22.61±1.30	22.72±0.81
*α-tub*	19.49±1.02	20.03±0.46	19.47±1.05	18.87±1.27	19.74±1.12	19.56 ±0.47
*β-tub*	20.48±0.99	21.07±0.52	20.36±0.83	19.89±1.40	20.74±0.90	20.52±0.51
*EF1-α*	18.72±1.01	19.74±0.43	18.58±0.93	18.25±1.30	18.71±0.84	18.64±0.80
*RPL13A*	20.50±1.41	20.99±0.54	20.13±0.92	19.62±1.97	20.68±1.48	21.30±0.51
*β-αct*	19.63±1.12	20.58±0.42	18.83±0.77	19.58±1.35	19.66±1.29	19.54±0.82
*GADPH*	19.59±1.11	20.11±0.51	19.16±1.09	19.24±1.33	19.48±1.27	20.08±0.77

### Stability ranking of the candidate reference genes

Expression stability of the candidate reference genes were determined by geNorm, NormFinder and BestKeeper, which evaluated the stability ranking of each reference gene by using the Ct values across all sample sets. The stability ranking analyses done by the three methods were detailed below.

#### geNorm analysis

**Fig 2:**
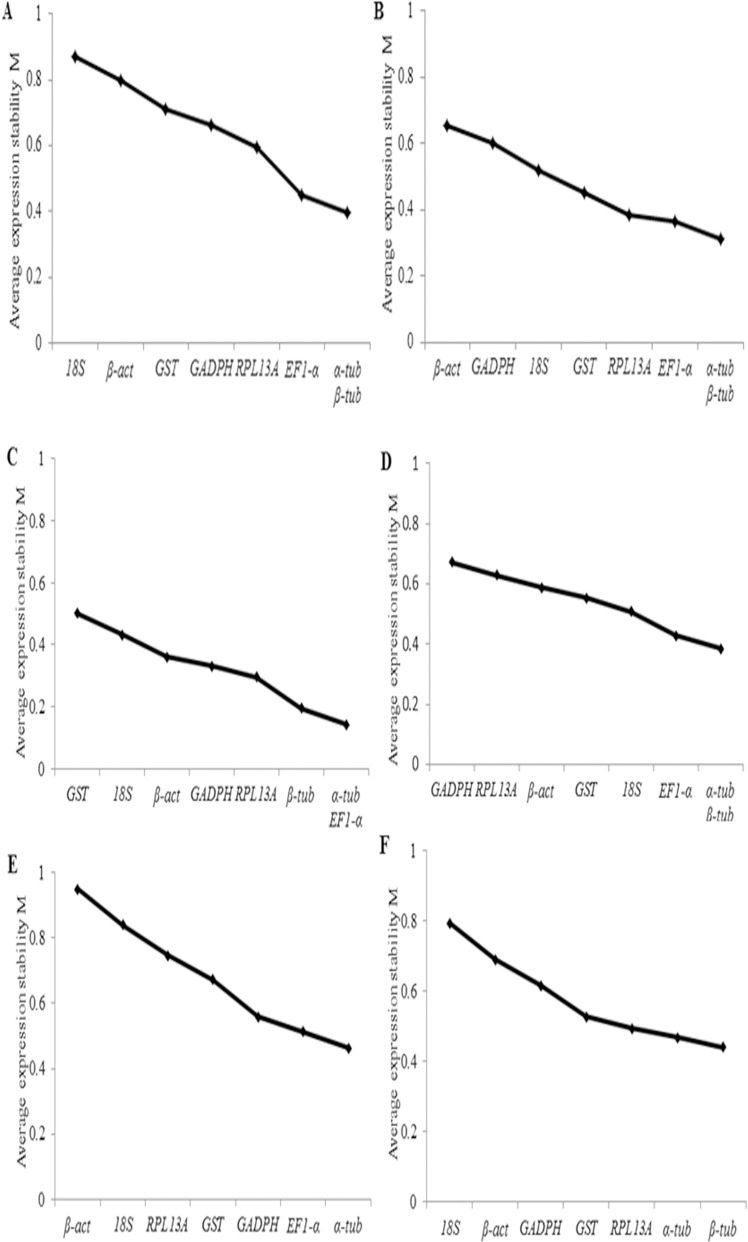
Stability rankings of eight candidate reference genes calculated by geNorm. The average expression stability values (M) of eight reference genes were plotted from the least stable (left) to the most stable (right) in (A) all samples, (B) control, (C) short-term cold exposure, (D) short-term heat exposure, (E) long-term cold exposure and (F) long-term heat exposure.

**Fig 3:**
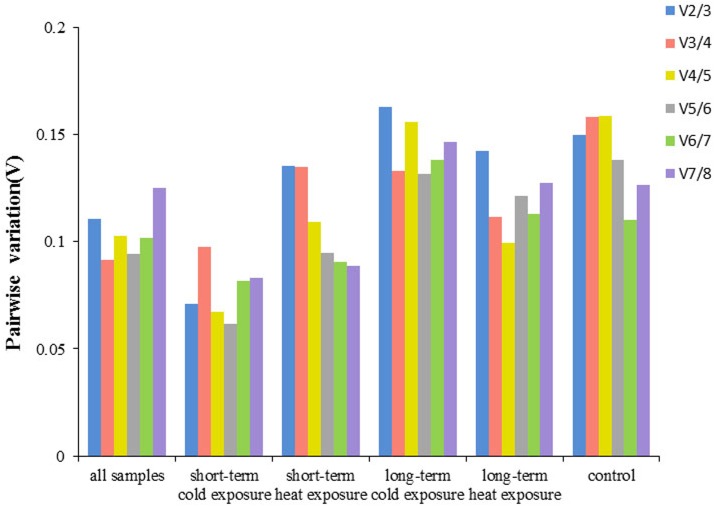
The pairwise variation (V) of eight candidate reference genes for an accurate normalization. The pairwise variation (Vn/Vn+1) was analyzed between normalization factors NFn and NFn+1 to determine the optimal number of references genes required for accurate normalization in MED. A threshold value of 0.15 was suggested for valid normalization.

The geNorm software is employed as a means for determining the average expression stability for all genes with a threshold expression stability M value of 0.5 to identify reference genes with stable expression. The geNorm ranking of the reference genes stability in all investigated samples are summarized in Table 3. The results suggested that *β-tub* was the most stable candidate reference gene in all 40 samples of MED, whereas *18S* was revealed less stability. Moreover, the two genes of *α-tub* and *β-tub* were identified as the most stably expressed genes in the control, short-term heat exposure and long-term cold exposure samples. While the geNorm analysis also indicated that *β-tub* and *EF1-α* were the most stably expressed genes in short-term cold exposure samples and long-term heat exposure samples (Fig 2). Furthermore, the traditional reference genes, such as *β-act*, *18S*, *GAPDH* did not exhibit high stability and even showed great variation under some conditions. Although most studies had used only one gene as an internal control for normalization, the use of two or more reference genes for normalization might produce more reliable results. To determine the optimal number of reference genes needed for normalization, geNorm also calculated the pairwise variation (Vn/n+1) between the two sequential normalization factors. The result provided a V2/3 below the cut off value of 0.15 indicating that two reference genes were best sufficient for normalization in all experimental conditions (Fig 3).

**Table 3:** Expression stability values of eight candidate reference genes calculated using geNorm.

	Stability deviation					
Gene name	All samples	Short-term cold exposure	Short-term heat exposure	Long-term cold exposure	Long-term heat exposure	Control
*18S*	1.10	0.59	0.68	1.14	1.10	0.73
*GST*	0.88	0.71	0.77	0.97	0.68	0.58
*α-tub*	0.75	0.38	0.58	0.84	0.71	0.61
*β-tub*	0.69	0.34	0.59	0.75	0.64	0.56
*EF1-α*	0.76	0.37	0.63	0.75	0.70	0.59
*RPL13A*	0.86	0.46	0.75	1.05	0.66	0.59
*β-αct*	1.06	0.53	0.66	1.28	0.97	0.82
*GADPH*	0.87	0.53	0.80	0.83	0.88	0.79

#### NormFinder analysis

The NormFinder analyzes gene expression variation for all eight candidate reference genes by calculating SD values, and the lowest SD value indicates the highest expression stability. The results are given in Table 4. This analysis method identified that *EF1-α* was the best optimal internal control genes in almost all of the samples and conditions, with the exception of the short-term cold exposure samples showed *β-tub* and *RPL13A* were the most stable genes (Table 4).

**Table 4:** Expression stability values of eight candidate reference genes calculated using NormFinder.

	Stability deviation					
Gene name	All samples	Short-term cold exposure	Short-term heat exposure	Long-term cold exposure	Long-term heat exposure	Control
*18S*	0.25	0.27	0.26	0.41	0.44	0.25
*GST*	0.31	0.25	0.19	0.28	0.44	0.27
*α-tub*	0.28	0.30	0.26	0.23	0.27	0.14
*β-tub*	0.18	0.09	0.16	0.14	0.19	0.18
*EF1-α*	0.12	0.14	0.12	0.12	0.10	0.12
*RPL13A*	0.22	0.09	0.20	0.19	0.21	0.18
*β-αct*	0.30	0.17	0.21	0.33	0.24	0.15
*GADPH*	0.27	0.21	0.18	0.37	0.32	0.26

#### BestKeeper analysis

BestKeeper analyzes the stability of candidate reference genes based on the coefficient of variance and the SD of the Ct values, by using the average Ct value of each duplication reaction [65]. Reference genes are identified as the most stable genes exhibited the lowest SD, and genes with SD less than 1 were considered acceptable [66]. As shown in Table 5, *β-tub* was the most stably expressed genes with the lowest SD values in all samples, short-term heat exposure and the control samples (0.69, 0.58 and 0.24, respectively). For both short- and long-term cold exposure samples, *GADPH* was the most stable gene with lowest SD values 0.33 and 0.91, respectively. While *EF1-α* was most stable in long-term heat exposure samples with the value of 0.68. The results of BestKeeper analysis showed little differences from those obtained from geNorm and Normfinder.

**Table 5:** Expression stability values of eight candidate reference genes calculated using BestKeeper.

	Stability deviation					
Gene name	All samples	Short-term cold exposure	Short-term heat exposure	Long-term cold exposure	Long-term heat exposure	Control
*18S*	1.03	0.67	0.73	1.03	1.62	0.58
*GST*	1.10	0.61	0.90	1.24	1.11	0.50
*α-tub*	0.74	0.34	0.85	0.98	0.82	0.37
*β-tub*	0.69	0.38	0.58	1.00	0.72	0.24
*EF1-α*	0.81	0.34	0.67	0.94	0.68	0.52
*RPL13A*	1.03	0.40	0.66	1.34	1.01	0.40
*β-αct*	0.87	0.35	0.63	1.09	0.76	0.68
*GADPH*	0.97	0.33	0.91	0.91	1.10	0.60

#### Validation of selected reference genes using a real case study

**Fig 4:**
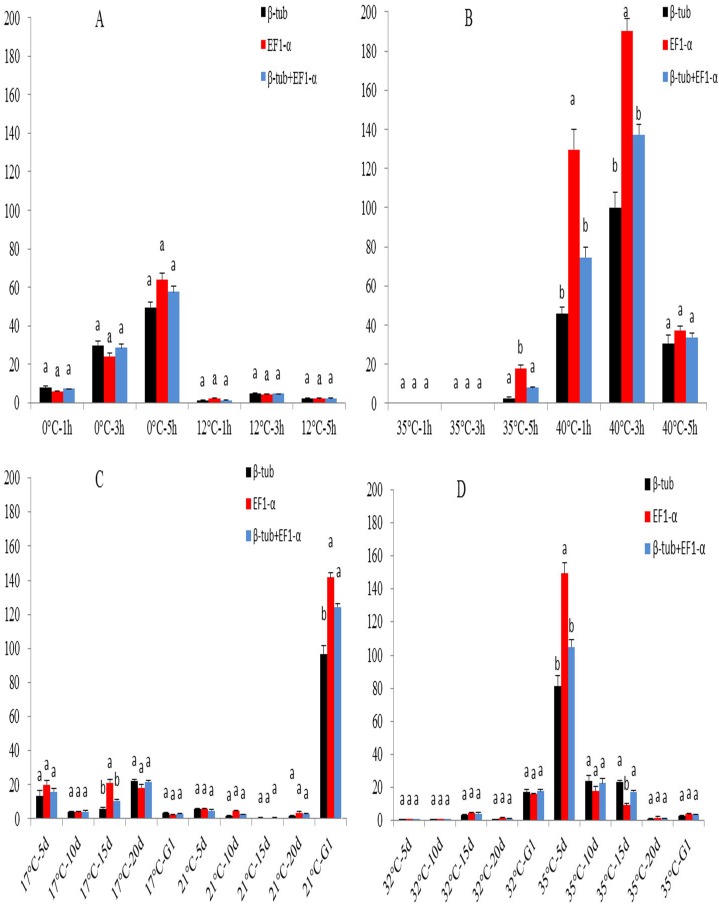
Normalized expression of the TRP under temperature stress using both single reference genes and gene combinations. Results are represented as mean ± SD. Different letters (a, b) shows the statistical difference (P <0.05) among the normalization strategies.

To evaluate the selected reference genes, we examined the expression patterns of *TRP* gene after various temperature stresses. The expression of *TRP* gene was normalized by both each of the selected stable genes (*β-tub* or *EF1-α*) and recommended gene combinations (*β-tub* + *EF1-α*). The expression of *TRP* normalized by *EF1-α* was significantly overrated at 40°C after 1h and 3h exposure (Fig 4B), at 17°C after 20 d exposure (Fig 4C) and at 35°C after 5 d exposure (Fig 4D), compared to the results obtained by *β-tub* and *β-tub* + *EF1-α* as internal control, whereas the expression of *TRP* normalized by *β-tub* was significantly underestimated at 21°C after the time of G0 to G1 exposure (Fig 4C) and at 35°C after 15 d exposure (Fig 4D), compared to the results obtained by *EF1-α* and *β-tub* + *EF1-α* as internal control. Consequently, the results suggested that the use of single reference gene may significantly influence the quantification of gene expression, while multiple reference genes are preferred to minimize possible errors.

## Discussion

Lately focus of biological invasions research has been directed towards to understand the mechanisms of quick adaptation to rising global temperature [31–33, 39, 67]. In particular, investigating the molecular mechanisms of thermotolerance during biological invasions can help to understand the ecological adaptation of invasive alien species, and further offer a great opportunity to dissect how organisms cope with rapidly changing environments in the wild. The most well-characterized molecular responses to temperature stresses are the variation of gene expression levels [68]. Furthermore, qRT-PCR is a preferred method for reliable quantification of gene expression levels. In qRT-PCR, normalization the gene expression levels of reference gene is critical for obtaining accurate and reliable relative quantification results [16, 69]. Rarely a reference gene could be non-differentially expressed across different experimental conditions [70]. For this, we used qRT-PCR to validate suitable reference genes for gene expression research under varied temperatures.

In the present study, eight candidate reference genes were carried out to test their efficacy under different thermal stresses in MED. Three different algorithms statistical models were applied to our data to providing a robust screening approach. It was assumed that a comparison of different mathematical models would allow reliable evaluation and avoid the selection of co-regulated transcripts [41]. The results showed that the top three reference genes determined by NormFinder were *EF1-α*, *RPL13A* and *β-tub*, whereas in GeNorm analysis methods, *β-tub*, *EF1-α* and *α-tub* were identified as the three most stable genes, additionally the top three reference genes produced by BestKeeper were not the all the same according to different experimental sets. Meanwhile, the results by all applied methods demonstrated that the traditional reference genes, such as *β-act*, *18S* and *GAPDH* did not always exhibit high stability and even showed great variation under some conditions. Slightly inconsistencies among these three algorithms were expected since different analytical principles are used by each of them as mentioned previously in “Materials and methods”. Although different best single reference gene or gene combinations were yielded among the three algorithms for all samples, the comprehensive ranking order for most group samples showed that *β-tub*, *EF1-α* and *α-tub* were nearly the top three most stable reference genes, demonstrating the suitability of the identified reference genes under various temperature stresses in MED.

*β-act* is a cytoskeletal protein and part of the contractile apparatus involving in a number of cellular processes [71], assumed to be moderately abundant expression [25]. In several insects, *β-act* has been top-ranked as a reference gene in expression studies under short-term thermal stresses in *Ditylum brightwellii* [72], *C*. *striatus* [52], *T*. *citricida* [53], *Sesamia inferens* [73]. A striking finding of this study was that the traditionally preferred reference gene *β-act* was highly variable and was ranked last one in most samples in our study, but not in the short-term temperature samples. As many previous studies had revealed that *β-act* were challenged for their suitability as the internal controls on *Leptinotarsa decemlineata* [74], *B*. *tabaci* [19], *Agrilus planipennis* [75] and *Tribolium castaneum* [76]. Maybe it was due to that mild heat stress might lead to *β-act* filaments reorganized into stress fibers, and extreme high temperature treatment could lead to the disorganized of *β-act* [77]. Consequentially, the expression level of *β-act* was instable under various temperature stresses.

The expression stability of *GAPDH* was moderated among all sample sets in this study, and the results were inconsistent with other studies, which identifying *GAPDH* as the most stable gene under thermal stresses in *P*. *citri* [55], *D*. *brightwellii* [72] and *C*. *striatus* [52]. Moreover, recent studies suggested that the expression of *GAPDH* gene varied considerably depending upon developmental stage, tissue, experimental condition and species [19, 53, 60], therefore it is a controversial gene. Hence, our results would not recommend *GAPDH* as a suitable reference gene for MED exposed to thermal stresses.

In contrast, our results showed *EF1-α* exhibited stable expression with respect to the most commonly used reference genes among various temperature stresses. *EF1-α* is involved in protein synthesis and is widely used as a normalizer in insects. For example, *EF1-α* appeared as the most stable genes under different developmental stages and tissues in *L*. *decemlineata* [74], *A*. *planipennis* [75], *Aphis glycines* [59] and *B*. *tabaci* [19]. Additionally, *EF1-α* also showed the highest stability in response to various abiotic factors such as in *Drosophila melanogaster* treated with heat-shock stress [25], as well as in *T*. *citricida* treated with starvation and UV irradiation stress [47].

*β-tub* was surprisingly identified as top-ranked reference genes in all sample sets. Similarly, *β-tub* was the most stable gene in *Ericerus pela* [78] and *T*. *citricida* [53] under temperature treatments, *T*. *armoricanus* under dietary treatments [58]. Thus, all these results suggested that each experiment should investigate a normalizer reference gene for a specific requirement and condition rather than adopting reference gene from other studies.

In the present study, we used the two most stable selected reference genes (*β-tub* and *EF1-α*) in whitefly under temperature stress, to normalize the expression of TRP separately. It showed significant different results at 40°C after 1h and 3h exposure, at 17°C after 20 d exposure, at 35°C after 5 d and 15d exposure and at 21°C after the time of G0 to G1 exposure (Fig 4). The results indicated that even using a single most stable reference gene after assessment, it may also mislead the explanation of expression analysis. As a result, to obtain reliable and accurate results, multiple reference genes should be used in quantitative gene expression studies.

## Conclusion

In summary, our work was the first in-depth study to identify and validate reference genes for qRT-PCR data normalization under comprehensive temperature stresses in MED. The present study suggested that *β-tub* and *EF1-α* had the greatest stability across various thermal treatments. *18S*, *GADPH* and *β-act* were the least stable gene suggesting that it should be not used as a reference gene to study gene expression under various thermal treatments in MED. In recent years, it has become clear that the expression stability of a putative reference gene has to be verified before each qRT-PCR experiment under all the experimental conditions. Meanwhile, a single reference gene is generally not enough to obtain reliable and accurate results and hence multiple reference genes should be used in quantitative gene expression studies. The present results would provide the compressive information of reference genes to study gene expression in MED and other invasive insects. Further, it will help to understand the underlying thermotolerance molecular mechanisms in invasive insects.

## Supporting information

S1 Fig Dissociation curve. (TIF)

S1 File Ct values of all samples. (PDF)
