# Supplementary figures and images for: Selection and validation of reference genes for qRT-PCR analysis during biological invasions: The thermal adaptability of Bemisia tabaci MED
Source: PLoS One. 2017 Mar 21;12(3):e0173821. doi: 10.1371/journal.pone.0173821 (PMC5360248; doi:10.1371/journal.pone.0173821)

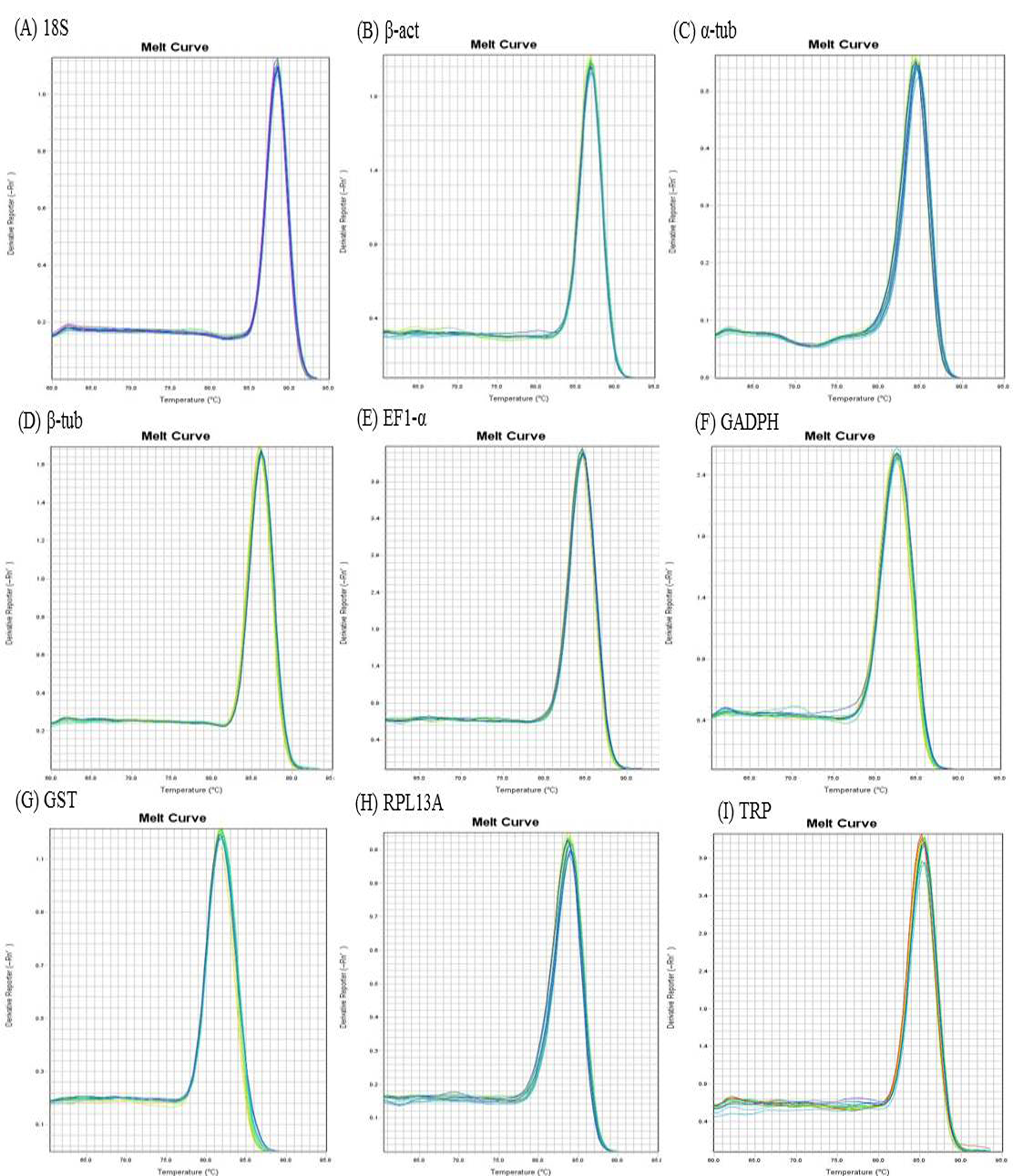

Supplement: S1 Fig — (TIF) [file pone.0173821.s001.tif]
