# Supplementary material for: Selection and validation of reference genes for qRT-PCR analysis during biological invasions: The thermal adaptability of Bemisia tabaci MED
Source: PLoS One. 2017 Mar 21;12(3):e0173821. doi: 10.1371/journal.pone.0173821 (PMC5360248; doi:10.1371/journal.pone.0173821)
Supplement: S1 File — (PDF) [file pone.0173821.s002.pdf]

(A) Ct values of short-term cold thermal stresses samples

Gene name	0 ℃			12 ℃		
	1h	3h	5h	1h	3h	5h
18S	10.93 ±1.12	11.39 ±0.73	10.14 ±0.23	9.79 ±0.85	9.50 ±0.61	8.90 ±0.15
GST	23.75 ±0.36	23.49 ±0.44	22.51 ±0.37	22.80 ±0.05	23.57 ±0.29	21.27 ±1.03
α-tub	20.36 ±0.75	20.66 ±0.34	20.06 ±0.51	19.49 ±0.48	20.08 ±0.38	19.54 ±1.05
β-tub	21.24 ±0.72	21.68 ±0.58	21.43 ±0.36	20.78 ±0.15	21.07 ±1.13	20.21 ±0.39
EF1-α	19.89 ±1.03	20.38 ±0.82	19.96 ±0.19	19.33 ±0.29	19.64 ±0.11	19.23 ±1.58
RPL13A	21.52 ±0.23	21.43 ±0.41	21.23 ±1.50	20.07 ±0.03	20.75 ±1.18	20.93 ±0.60
β-αctin	21.04 ±0.23	20.80 ±1.12	20.79 ±0.23	20.04 ±0.65	20.07 ±0.38	20.74 ±0.59
GADPH	20.10 ±0.08	20.51 ±1.48	20.70 ±1.11	19.22 ±0.26	20.11 ±0.28	20.04 ±0.36
TRP	28.22 ±0.13	26.04 ±0.10	25.12 ±0.09	26.25 ±0.13	27.54 ±0.06	27.99 ±0.15

(B) Ct values of short-term heat thermal stresses samples

Gene name	35 ℃			40 ℃		
	1h	3h	5h	1h	3h	5h
18S	9.78 ±1.01	10.27 ±1.06	9.92 ±1.36	8.88 ±1.12	9.94 ±0.89	8.70 ±1.19
GST	21.10 ±0.48	22.84 ±0.12	22.02 ±0.97	20.46 ±0.14	22.58 ±0.28	20.46 ±1.23
α-tub	19.77 ±0.26	20.09 ±0.50	19.62 ±0.70	17.94 ±0.00	20.81 ±0.73	18.56 ±1.34
β-tub	20.49 ±0.32	20.93 ±2.03	20.49 ±0.42	18.89 ±0.12	21.28 ±0.16	20.09 ±0.99
EF1-α	19.16 ±0.79	18.57 ±0.49	18.51 ±0.64	17.37 ±0.28	20.01 ±0.14	17.87 ±0.92
RPL13A	21.26 ±0.16	20.90 ±0.83	20.20 ±0.84	18.68 ±0.46	20.10 ±0.58	19.63 ±0.4
β-αctin	19.41 ±0.06	18.97 ±0.38	19.72 ±0.03	18.03 ±0.03	19.09 ±0.35	17.78 ±1.03
GADPH	20.51 ±0.72	18.60 ±0.93	19.39 ±0.45	17.97 ±0.35	20.32 ±0.34	18.18 ±0.18
TRP	30.30 ±0.24	32.87 ±0.1	28.94 ±0.31	25.22 ±0.10	24.69 ±0.28	24.95 ±0.20

(C) Ct values of control samples

Gene name	Control (26 ℃)							
	1h	3h	5h	5d	10d	15d	20d	F1
18S	11.95±0.87	10.66±0.39	10.21±0.31	10.06±0.37	11.40±0.27	10.25±0.22	10.29±0.20	9.64±0.34
GST	23.38±0.81	23.57±0.04	22.87±0.08	21.26±0.01	23.47±0.03	22.97±0.40	22.14±0.01	22.13±0.08
α-tub	19.71±0.19	19.88±002	20.19±0.02	19.39±0.11	19.93±0.05	18.69±0.11	19.23±0.08	19.47±0.10
β-tub	21.17±0.14	20.89±0.11	20.70±0.13	20.38±0.13	20.79±0.08	20.07±0.88	19.55±0.01	20.60±0.08
EF1-α	19.67±0.82	19.46±0.17	19.24±0.05	18.51±0.11	18.49±0.05	17.89±0.15	17.30±0.06	18.57±0.02
RPL13A	21.86±0.17	21.66±0.06	21.95±0.18	21.04±0.06	21.15±0.09	21.11±0.05	20.39±0.16	21.21±0.02
β-actin	20.21±0.43	20.58±0.30	19.90±0.03	19.51±0.12	18.45±0.07	20.19±0.12	19.05±0.17	18.45±0.06
GADPH	20.03±1.05	20.57±0.11	20.88±0.12	19.25±0.06	20.40±0.03	20.87±0.41	19.90±0.03	18.72±0.04
TRP	30.86±0.05	30.79±0.08	30.89±0.05	26.70±0.03	29.12±0.07	28.43±0.03	28.16±0.05	27.34±0.10

(D) Ct values of long-term cold thermal stresses samples

Gene name	17 ℃					21 ℃				
	5d	10d	15d	20d	F1	5d	10d	15d	20d	F1
18S	8.67±0.70	9.16±1.10	8.72±0.91	8.91±0.56	11.36±0.57	8.50±0.37	9.96±0.45	11.10±0.54	8.48±0.36	9.28±0.83
GST	18.73±0.13	22.50±0.72	19.81±0.35	20.42±0.19	23.15±60	19.16±0.16	22.73±0.31	22.40±1.48	20.37±0.81	20.27±0.08
α-tub	17.80±0.2	20.01±0.27	19.20±0.33	17.07±0.26	19.73±0.34	16.64±0.03	20.00±0.30	20.05±0.46	19.47±0.77	18.73±0.32
β-tub	18.01±0.22	21.18±0.98	20.40±1.00	18.53±0.98	21.70±0.48	17.63±0.37	20.65±0.43	21.06±1.48	20.05±0.56	19.69±1.14
EF1-α	16.98±0.26	19.62±0.66	18.66±0.38	16.88±0.12	19.90±0.54	16.47±0.20	18.54±0.19	19.90±0.72	18.22±0.73	17.27±0.68
RPL13A	16.70±0.50	21.02±0.54	19.86±1.61	18.90±0.81	22.04±0.13	16.53±0.18	20.15±0.95	22.13±1.28	20.37±0.50	18.51±0.41
β-actin	19.02±0.30	20.47±0.27	20.18±0.87	18.89±0.60	18.90±0.29	17.78±0.88	19.73±0.42	21.52±0.71	21.52±0.16	17.79±0.94
GADPH	17.81±0.07	20.57±1.64	19.33±0.94	18.44±0.50	20.62±0.16	16.84±0.61	19.00±1.76	20.51±0.34	20.57±0.29	18.69±0.35
TRP	26.39±0.28	29.02±0.16	26.20±0.25	25.00±0.04	28.37±0.39	25.04±0.25	28.52±0.16	29.22±0.18	25.54±0.10	25.08±0.14

(E) Ct values of long-term heat thermal stress samples

Gene	32 ℃					35 ℃				
name	5d	10d	15d	20d	F1	5d	10d	15d	20d	F1
18S	9.38±0.19	13.67±0.42	9.53±0.41	10.46±0.13	9.28±0.66	9.96±0.28	12.31±0.44	13.04±0.14	12.74±0.26	13.01±0.82
GST	20.47±0.48	23.55±0.30	21.17±0.44	22.96±0.22	21.39±0.52	21.88±0.01	23.07±0.30	23.57±0.30	23.79±0.18	24.30±0.18
α-tub	17.61±0.19	20.70±0.07	19.66±0.66	19.80±1.05	19.02±0.23	19.00±0.38	19.34±0.15	20.04±0.30	20.55±0.24	21.72±0.30
β-tub	19.14±0.34	21.28±0.35	20.48±0.30	20.41±0.09	19.60±1.01	20.48±1.42	21.01±0.10	21.51±0.18	21.43±0.39	22.04±1.00
EF1-α	17.18±0.39	19.67±0.45	18.44±0.42	18.88±0.14	18.12±0.28	17.96±1.08	18.47±0.64	19.15±0.75	19.79±0.29	19.49±0.23
RPL13A	17.80±0.40	21.42±0.08	19.87±1.21	22.73±0.14	19.66±0.55	19.65±0.33	20.28±0.10	21.72±0.18	21.71±0.40	21.97±0.45
β-αctin	17.93±0.80	19.38±0.14	20.64±0.12	20.92±0.18	18.17±0.36	19.46±0.18	18.04±0.72	21.64±0.23	20.00±0.34	20.38±0.15
GADPH	17.66±0.52	19.15±0.49	18.39±0.27	18.55±0.44	18.76±0.35	18.88±0.27	20.40±0.22	21.27±0.32	20.61±0.84	21.14±0.16
TRP	32.64±0.06	31.06±0.14	28.56±0.09	26.60±0.12	25.17±0.04	25.29±0.15	24.47±0.63	22.92±0.22	26.73±0.31	28.41±0.10
